# A small *Acinetobacter* plasmid carrying the *tet39* tetracycline resistance determinant

**DOI:** 10.1093/jac/dkv293

**Published:** 2015-09-27

**Authors:** Mohammad Hamidian, Kathryn E. Holt, Derek Pickard, Ruth M. Hall

**Affiliations:** 1 School of Molecular Bioscience, The University of Sydney, NSW 2006, Australia; 2 Department of Biochemistry and Molecular Biology, Bio21 Molecular Science and Biotechnology Institute, The University of Melbourne, Melbourne, VIC, Australia; 3 Wellcome Trust Sanger Institute, Hinxton, Cambridge, UK

Sir,

Tetracycline can still be used for treatment of infections caused by Gram-negative organisms that lack resistance genes. In *Acinetobacter baumannii* tetracycline resistance is often caused by *tetA*(A) and *tetA*(B), which encode efflux pumps,^1,2^ and occasionally by *tet*(M), which encodes a ribosomal protection protein*.*^1^ In addition, a novel determinant, *tet39* (*tetA39*-*tetR39*), was found in tetracycline-resistant *Acinetobacter* strains recovered from freshwater fish farms in Denmark^3^ and Thailand^4^ and from a clinical *Acinetobacter calcoaceticus*/*baumannii* complex isolate recovered from human urine in 1986.^3^ The *tetA39* gene encodes an efflux pump that was reported to confer resistance to tetracycline but not minocycline.^3^

In strains belonging to global clone 1 (GC1), the *tetA*(A) gene is part of the AbaR resistance island^5^ while in GC2 isolates the *tetA*(B) gene is found in an AbGRI1 resistance island.^6^ However, there is little information about the tetracycline resistance determinants and their context in strains that do not belong to the two major global clones. Here, we have examined the cause of tetracycline resistance in RCH52, a clinical multiply antibiotic-resistant *A. baumannii* strain recovered prior to 2010 in a Queensland hospital.

RCH52 was found to be resistant to ampicillin, ceftazidime, ticarcillin/clavulanic acid, imipenem, meropenem, streptomycin, spectinomycin, sulphonamides, trimethoprim, kanamycin, gentamicin and tetracycline. The genome of RCH52 was sequenced using the Illumina Hiseq platform and assembled as described previously,^7^ generating 60 contigs. RCH52 was ST729 (Institut Pasteur scheme), a novel single locus variant of ST3 (*rpoB4* in ST729 differs from *rpoB3* in ST3 by a single base pair) and RCH52 therefore belongs to the European clone III. To the best of our knowledge this is the first report of the European clone III in Australia. ResFinder 2.1 (https://cge.cbs.dtu.dk//services/ResFinder/) was used to identify resistance genes, and RCH52 contains *aphA1b*, *aacC2*, *aadA1*, *floR*, *cmlA1*, *arr-2*, *sul1*, *sul2*, *dfrA14*, *oxa10*, *bla*_TEM_, *oxa23* (encoding a variant of OXA-23 differing by four amino acids) and the *tet39* determinant, accounting for the resistances observed. The *comM* gene is uninterrupted and ISAba1 was not found upstream of the *ampC* gene.

The *tet39* determinant was found on an 11 kb contig that was shown to be circular using PCR followed by sequencing. The plasmid carrying *tet39*, named pRCH52-1 (GenBank accession number KT346360; Figure 1), is 11146 bp. Plasmid DNA was isolated and electroporated into *A. baumannii* ATCC 17978, which is tetracycline susceptible. Transformants were selected on L-agar supplemented with 20 mg/L tetracycline (transformation frequency = 4.5 × 10^7^ transformants/μg of DNA). None of the transformants grew on L-agar containing other antibiotics that RCH52 was resistant to, indicating that only the tetracycline resistance had transferred into the recipient cells. Primers tet39-F (5′-GCAGCTAATGCCCATACCAT-3′) and tet39-R (5′-GCCTTTTGCGTTGTTACCAT-3′) were designed to amplify a 219 bp internal fragment of the *tetA39* gene. This PCR generated the expected product for all of the transformants tested. Although it was reported that *tet39* does not confer resistance to minocycline,^3^ transformants tested here exhibited reduced susceptibility to minocycline, with inhibition zone diameters of 18 mm compared with 26 mm for ATCC 17978.

**Figure 1.**
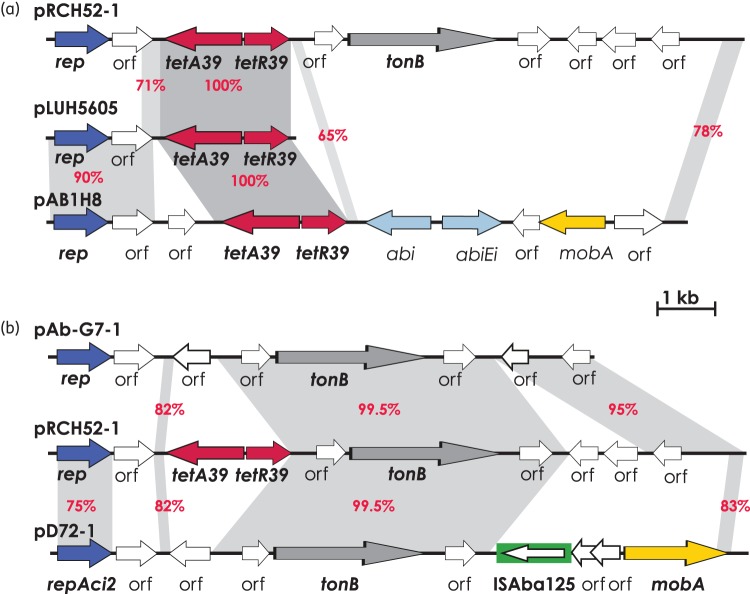
Linearized map of pRCH52-1 compared with other plasmids. (a) Comparison of the original *tet39* region found in pLUH5605 with the corresponding region found in pRCH52-1 and pAB1H8. (b) pRCH52-1 compared with the cryptic plasmids pAb-G7-1 and pD72-1 seen in GC1 and GC2 strains, respectively. Arrows indicate the extent and direction of genes and ORFs. The *tetA* gene and *tetR* genes of *tet39* are shown in red and *rep* genes are coloured blue. The *tonB* gene encodes a TonB-dependent transporter homologue. The green box indicates ISAba125 and the arrow inside represents the transposase gene. The extents of regions with significant DNA identities are shown in grey and red numbers represent DNA identities. A scale bar is also shown. The picture is drawn to scale from the following GenBank entries: pRCH52-1, GenBank accession number KT346360; pAb-G7-1, GenBank accession number KJ586856; pD72-1, GenBank accession number KM051986; pLUH5605, GenBank accession number AY743590; and pAB1H8, GenBank accession number ANNC01000048. This figure appears in colour in the online version of *JAC* and in black and white in the print version of *JAC*.

The original study showed that *tet39* was located on plasmids but only a 3727 bp fragment of one plasmid (named here pLUH5605) was sequenced (GenBank accession number AY743590). This sequence, the only complete *tet39* sequence found in the GenBank non-redundant database, includes *tetA39*, *tetR39* and two ORFs (Figure 1a), one of which encodes a replication initiation protein.^3^ Only 2360 bp of the pLUH5605 sequence was present in pRCH52-1 (Figure 1a). To explore the distribution of the *tet39* determinant in *Acinetobacter* strains, the whole genome sequence database of NCBI was explored using the sequences of pRCH52-1 and pLUH5605. Twenty-one *Acinetobacter* strains belonging to different species were found to contain *tet39* (Table S1, available as Supplementary data at *JAC* Online). However, the *tet39* region appears to be in different contexts in all but one *A. baumannii* strain, AB1H8 (GenBank accession number ANNC01000048). AB1H8 appears to include a plasmid that contains a Rep that is 97% identical in terms of amino acids to the pLUH5605 RepA (Figure 1a). Hence, discrete boundaries surrounding *tet39* were not found.

pRCH52-1 encodes a replication initiation protein Rep that belongs to the Rep_3 superfamily (pfam01051) and differs from the Rep found in pLUH5605 by 43%. The pRCH52-1 Rep is identical to the Rep protein found in a strain belonging to the *A. calcoaceticus*/*baumannii* complex (NCBI Reference Sequence number WP_000845850). Thereafter, the closest Rep is RepAci7 (GenBank accession number GU978996), with 96% amino acid identity.

pRCH52-1 also encodes a TonB-dependent transporter homologue. These outer membrane proteins bind and transport siderophores, vitamin B_12_, nickel complexes and carbohydrates.^8^ This gene is also present in several cryptic plasmids of *A. baumannii*,^6,7,9,10^ including pAb-G7-1 and pD72-1 from GC1 and GC2 strains, respectively. However, the amino acid sequence of Rep encoded by pRCH52-1 differs from those in pAb-G7-1 (GenBank accession number KJ586856) and pD72-1 (GenBank accession number KM051986) by 24% and 21%, indicating that the *tonB* gene, which is a potential virulence determinant, is widely distributed.

The *tet39* determinant is widespread in *Acinetobacter* species. It has also been found in other species of Gram-negative and Gram-positive bacteria recovered from a polluted Nigerian river.^11^ Its location on plasmids in clinical isolates would facilitate the spread of tetracycline resistance amongst *Acinetobacter* strains, leading to further restriction of treatment options.

## Funding

This study was supported by NHMRC Project Grant 1026189 and Wellcome Trust grant number 098051. M. H. was supported by NHMRC Project Grant 1026189. K. E. H. was supported by an NHMRC Fellowship (no. 1061049).

## Transparency declarations

None to declare.

## Supplementary data

Table S1 is available as Supplementary data at *JAC* Online (http://jac.oxfordjournals.org/).

Supplementary Data
