# Supplementary material for: A small Acinetobacter plasmid carrying the tet39 tetracycline resistance determinant
Source: J Antimicrob Chemother. 2015 Sep 27;71(1):269–71. doi: 10.1093/jac/dkv293 (PMC4681370; doi:10.1093/jac/dkv293)
Supplement: Supplementary Data [file supp_71_1_269__index.html]

A small Acinetobacter plasmid carrying the tet39 tetracycline resistance determinant — A small Acinetobacter plasmid carrying the tet39 tetracycline resistance determinant — Supplementary Data 

# A small *Acinetobacter* plasmid carrying the *tet39* tetracycline resistance determinant

## Supplementary Data

Supplementary Data

- Supplementary Data - Doc file
