# Supplementary material for: A small Acinetobacter plasmid carrying the tet39 tetracycline resistance determinant
Source: J Antimicrob Chemother. 2015 Sep 27;71(1):269–71. doi: 10.1093/jac/dkv293 (PMC4681370; doi:10.1093/jac/dkv293)
Supplement: Supplementary Data [file supp_dkv293_dkv293supp.doc]

**Supplementary data**

**Table S1.** Properties of *Acinetobacter* genomes containing the *tet39* region found in WGS database

| Species | Strain | Global Clone | ST ^a^ | Isolation date | Country | Source | Contig no. | Contig size | Matching sequence^b^ | | GenBank accession no. |
| --- | --- | --- | --- | --- | --- | --- | --- | --- | --- | --- | --- |
|  |  |  |  |  |  |  |  |  | Left | Right |  |
| *A. baumannii* | MRSN16875 | na ^c^ | 10 | 2011 | Alaska | Sputum | 56 | 3755 | 771 | 33 | LAZK01000000 |
| *A. baumannii* | MRSN16897 | na | 10 | 2011 | Alaska | Blood | 55 | 5760 | 771 | 33 | LAYM01000000 |
| *A. baumannii* | NIPH 201 | na | 38 | nk ^d^ | USA | nk | 18 | 2720 | 263 | 558 | APQV01000000 |
| *A. baumannii* | CI86 | na | 25 | 2005 | Iraq | Wound | 17 | 8521 | 489 | 82 | AVOB01000000 |
| *A. baumannii* | AB4A3 | - | 255 | 2006 | Hong Kong | Wound | 82 | 7968 | 78 | 222 | AOLU01000000 |
| *A. baumannii* | AB1H8 | 2 | 2 | 2005 | Hong Kong | Sputum | 48 | 10246 | 78 | 209 | ANNC01000000 |
| *A. baumannii* | M2 | na | 16 | 2009 | Malaysia | nk | 82 | 3607 | 78 | 84 | LAKP01000000 |
| *A. baumannii* | 1999BJAB11 | 2 | 2 | 1999 | China | Sputum | 66 | 31300 | 212 | 69 | JSDB01000000 |
| *A. baumannii* | Naval-18 | na | 25 | 2006 | USA | Wound | 8 | 8422 | 489 | 82 | AFDA02000000 |
| *A. baumannii* | CI79 | na | 25 | 2005 | Iraq | Respiratory tract | 81 | 8523 | 489 | 82 | AVOD01000000 |
| *A. baumannii* | R1B | 1 | 1 | 2011 | Saudi Arabia | nk | 22 | 31788 | - | 82 | JICK01000000 |
| *A. baumannii* | 1 | na | - ^e^ | 2014 | Malaysia | Water | 24 | 5385 | 80 | 49 | JSUR01000000 |
| *A. baumannii* | 1 | na | - ^e^ | 2014 | Malaysia | Water | 53 | 6079 | 65 | 82 | JSAO01000000 |
| *A. baumannii* | 3390 | na | 10 | nk | USA | Perirectal | 47 | 1246 | - | - | JFER01000000 |
| *A. nosocomialis* | NIPH 386 | na | 410 | nk | USA | nk | 7 | 10249 | 196 | 82 | APPP01000000 |
| *A. gerneri* | KCTC 12415 | na | - ^f^ | nk | USA | WTP ^g^ | 129 | 3112 | 85 | 82 | BBLI01000000 |
| *A.gerneri* | MTCC 9824 | na | - ^f^ | nk | USA | WTP ^g^ | 73 | 7008 | 85 | 82 | ASYY01000000 |
| *A.gerneri* | DSM 14967 | na | - ^f^ | nk | Australia | WTP ^g^ | 3 | 9612 | 85 | 82 | APPN01000000| |
| *Acinetobacter* sp. | NIPH 899 | na | - ^f^ | nk | USA | nk | 88 | 2560 | - | 68 | APPE01000000 |
| *Acinetobacter* sp. | ver3 |  | - ^f^ | nk | Argentina | Hypersaline Water | 62 | 15875 | 76 | 82 | JFYL01000000 |
| *Acinetobacter* sp. | MDS7A | na | - ^f^ | nk | India | Dairy sludge | C64 | 3916 | 90 | 110 | AOTJ01000000 |

^a^ MLST, Institut Pasteur scheme.

^b^ number of bases flanking the *tet39* region matching pRCH52-1 relative to the stop codons of *tetA39* and *tetR39*. The surrounding sequence of pRCH52-1 starts matching the sequence of pG7-1, 46 bp on the left and 55 bp on the right relative to the stop codons of *tetA39* and *tetR39*.

^c^ not applicable

^d^ not known.

^e^ ST could not be assigned as the genome does not include the sequence of the allele(s) used in the MLST scheme.

^f^ ST could not be determined as the alleles had not been defined in the MLST scheme.

^g^ Wastewater treatment plant.
